# Characteristics of gut microbiota in people with obesity

**DOI:** 10.1371/journal.pone.0255446

**Published:** 2021-08-10

**Authors:** Mengmeng Duan, Yuezhu Wang, Qiang Zhang, Rong Zou, Min Guo, Huajun Zheng

**Affiliations:** 1 NHC Key Lab. of Reproduction Regulation (Shanghai Institute of Planned Parenthood Research), Fudan University, Shanghai, China; 2 Shanghai-MOST Key Laboratory of Health and Disease Genomics, Chinese National Human Genome Center at Shanghai, Shanghai, China; 3 Department of Obstetrics and Gynecology, Affiliated Hospital of Zunyi Medical College, Zunyi, China; State Key Laboratory for Diagnosis and Treatment of Infectious Diseases, CHINA

## Abstract

**Background:**

Obesity is the cause of cardiovascular diseases and other diseases, leading to increased medical costs, and causing a great burden to individuals, families and society. The prevalence of obesity is increasing and has become a global health problem. There is growing evidence that gut microbiota plays an important role in obesity. In this article, we revealed the differences in the gut microbiota between 21 people with obesity and 21 control subjects, and predicted the functional potential changes by 16S rRNA sequencing of the fecal bacteria of the subjects.

**Methods:**

The raw sequencing data of 21 healthy Beijing volunteers was downloaded from Microbial Genome Database System. Microbial 16S rRNA genes of 21 adults with obesity were sequenced on an Illumina MiSeq instrument and analyzed by using bioinformatics and statistical methods.

**Results:**

The diversity of gut microbiota in people with obesity decreased significantly. There were significant differences in gut microbiota between the Obesity and Control group at different levels. At the phylum level, *Firmicutes*, *Bacteroidetes*, *Actinobacteria* and *Fusobacteria* are significantly different between the Obesity and Control group. In people with obesity, the ratio of *Firmicutes*/*Bacteroidetes* decreased significantly. At the genus level, there were significant differences among the 16 major genera, of which four genera *Prevotella*, *Megamonas*, *Fusobacterium* and *Blautia* increased significantly in people with obesity, while the remaining 12 genera, *Faecalibacterium*, *Lachnospiracea_incertae_sedis*, *Gemmiger* and *Clostridium XlVa*, etc. decreased significantly. At the species level, nine species including *Bacteroides uniformis* and *Prevotella copri* had significant differences. Compared with the control group, subjects with obesity were abnormalities in 57 pathways, mainly in Carbohydrate metabolism and Lipid metabolism.

**Conclusions:**

Overall, our study revealed differences in the gut microbiota between people with obesity and control subjects, providing novel target for the treatment of individuals with obesity.

## Introduction

The most common method used to classify obesity is body mass index (BMI), which is calculated as weight divided by the square of height (kg/m^2^). When the BMI value is greater than or equal to 30 kg/m^2^, it is obese [1]. A number of studies have shown that the prevalence of obesity in different countries is increasing year by year [2–4]. In 2005, there were approximately 396 million adults with obesity in the world. By 2030, the number of adults with obesity may increase to 573 million [5]. The prevalence of childhood obesity has also increased significantly worldwide [6]. Hayes A et al. studied the relationship between childhood obesity and direct medical expenses, and found that their medical expenses were 1.62 times than that of normal-weight children [7]. Childhood obesity can also lead to high medical costs for a lifetime [8]. Obesity is a major cause of kidney and cardiovascular diseases [9]. Obesity will always be a serious health risk [1] and imposes a heavy burden on individuals and society. Therefore, it is necessary to study the causes of obesity and intervene to effectively reduce obesity.

A variety of factors including food choices, behavior, heredity and gut microbiota may contribute to obesity [10]. The choice of food directly affects calorie intake [10], and the daily consumption of sugary drinks increases the risk of obesity [11]. There is evidence that variations in the microbiota play a larger role in the pathogenesis of obesity [12]. The gut microbiota is important for human health, affecting the development of metabolic diseases and gastrointestinal diseases. Diet and the environment have an important impact on shaping the gut microbiota [13]. Newborns are born without gut microbiota and gradually form a stable gut microbiota structure at the age of 3–4 [14]. At present, the complete bacterial species of human gut microbiota is still uncertain. In addition to the 553 species previously cultured from the intestinal tract, Almeida A et al. also identified 1,952 candidate species that were not cultured [15]. *Bacteroidetes*, *Firmicutes*, and *Actinobacteria* are the three most abundant phyla in the intestine [14]. The gut microbiota of obesity and lean people is different, and the microbiota of people with obesity has an increased ability to get energy from their diet. Colonization of ‘obesity microbiota’ in sterile mice resulted in a significant increase in fat than colonization of ‘lean microbiota’ [16]. Intestinal anaerobic bacteria, including *Firmicutes* and *Bacteroidetes*, could hydrolyze carbohydrates that are indigestible in the gut, producing short-chain fatty acids (SCFAs) including acetate, propionate and butyrate, which have an effect on human health [17]. Free fatty acid receptor 3/G-protein coupled receptor 41 (FFAR3/GPR41) is the receptor for SCFAs, which is related to reduced food intake, increased energy consumption and expression of leptin hormone [18]. Therefore, anaerobic bacteria can inhibit obesity.

In order to study the characteristics of the gut microbiota of people with obesity, we compared the gut microbiota of 21 people with obesity from Shandong Province of China and 21 normal people from Beijing by 16S rRNA sequencing of fecal samples. The results of this study provide strong support for regulating gut microbiota to reduce obesity.

## Materials and methods

### Sample collection

Twenty-one adults with obesity were recruited from a gym of Jinan, Shandong Province (mean BMI 35.3, ranging from 31.4 to 49.5, 16 males, 5 females, with an average age of 35 years). All of them were not under dietary or medication control to lose weight, and did not take antibiotics for one month before fecal sample collection. Subject feces were collected and stored at -80 °C for subsequent analysis to reveal the differences in gut microbiota between people with obesity and control subjects. The study was approved by the Medical Ethical Committee of Shanghai Institute of Planned Parenthood Research (NO: PJ2019-17), and written informed consents were obtained from all subjects involved in this study. The raw sequencing data of 21 healthy Beijing volunteers (mean BMI 20.2, ranging from 16.5 to 25.4, 10 females, 11 males, with an average age of 26 years) was used as healthy control [19], which was sequenced using the same method as this study and downloaded from Microbial Genome Database System (http://data.mypathogen.org, ID = ICDC-20180224-143509).

### Genomic DNA extraction, PCR amplification and 16S rRNA gene sequencing

Total genomic DNA was extracted using QIAamp DNA Stool Mini Kit (QIAGEN). V3-4 region of 16S rRNA genes in people with obesity was amplified with primers 338F (5’-CCTACGGGNGGCWGCAG-3’) and 806R (5’-GACTACHVGGGTATCTAATCC-3’) using TransStart Fastpfu DNA Polymerase (TransGen). Cycling conditions were as follows: denaturation at 95°C for 2 min, 20 cycles of amplification (45 s at 95°C, 30 s at 55°C and 30 s at 72°C), extension 72°C for 5 min. Three repeat PCR amplifications of each sample were purified with AxyPrep DNA Gewendul Extraction kit (AXYGEN) and assessed by spectrophotometry (QuantiFluor-ST, Promega). The equivalent pooled 16S rRNA PCR amplicons were sequenced on an Illumina MiSeq instrument.

### Bioinformatics and statistical analysis

Raw paired FASTQ files were processed using Mothur (version 1.39.5) [20]. The following criteria were used to remove low quality sequences: containing ambiguous bases, the length shorter than 380bp, chimeric sequence, and contaminant sequence. After data normalization, the SILVA reference database [21] (V138) was used as reference for OTU (Operational taxonomic unit) identification under the threshold of 97% similarity. The taxonomic affiliation assignments were based on Ribosomal Database Project [22] at default parameter (80% threshold). Community richness, evenness and diversity analysis (ACE, Chao, Simpsonenven, Shannon, Simpson, and Good’s coverage) were performed using Mothur. Differences between obesity and control samples were assessed using Analysis of Molecular Variance (AMOVA) in Mothur. Microbiome functions were predicted using PICRUSt2 [23] through normalizing the 16S rRNA copy numbers. The taxonomy (OTU, genus, family and phylum) abundance differences and microbiome functional differences between the Obesity and Control groups were analyzed by STAMP [24] (*P*<0.05, difference between proportions >0.5 or ratio of proportion >1.2). The correlation coefficients of abundant genera were calculated using SparCC and visualized using Cytoscape version 3.8.2, and only correlations with SparCC > 0.35 or <-0.35 and P < 0.01 were included.

### Accession numbers

The sequence data have been submitted to the GenBank Sequence Read Archive (accession number PRJNA593870).

## Results

### Bacterial populations in Obesity and Control gut

**Fig 1:**
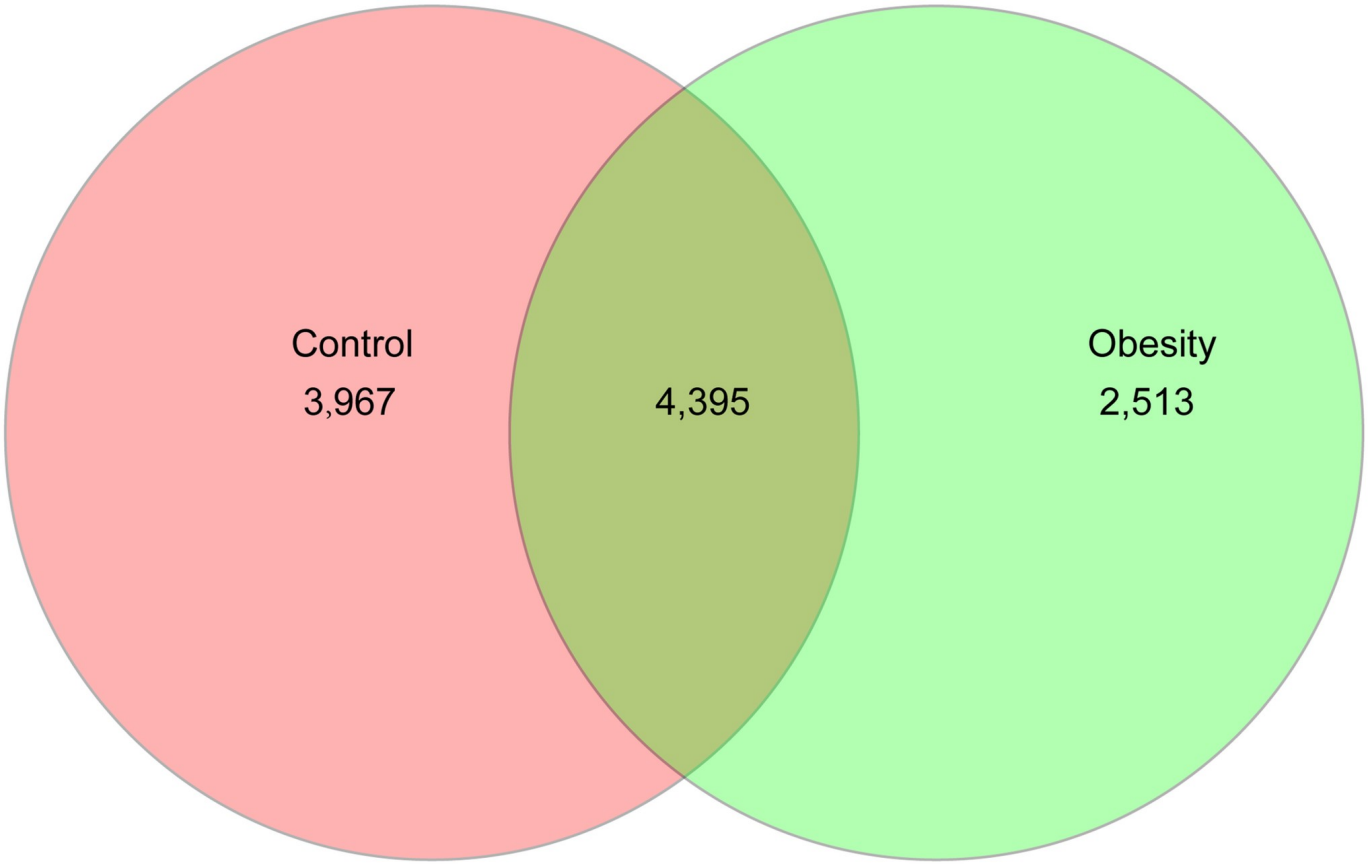
Venn chart illustrating the common and unique OTUs between Obesity and Control groups.

A total of 1,943,896 (39,477~ 59,690) high-quality sequences of 16S rRNA genes from 42 samples were obtained by high-throughput DNA sequencing. To normalize data avoiding statistical bias, 39,477 16S rRNA genes of each sample were chosen to calculate richness, evenness and diversity of the bacterial community at 97% similarity. After 42 samples were classified into two groups (Obesity and Control), a total of 10,875 OTUs were obtained. Of which 40.41% of OTUs were shared by the two groups, and the Obesity group had 1,454 OTUs less than the Control group (Fig 1) The Good’s coverage was over 99.88% for the two groups, indicating that the sequencing depth was sufficient for gut microbiota investigation of obesity and health people.

**Fig 2:**
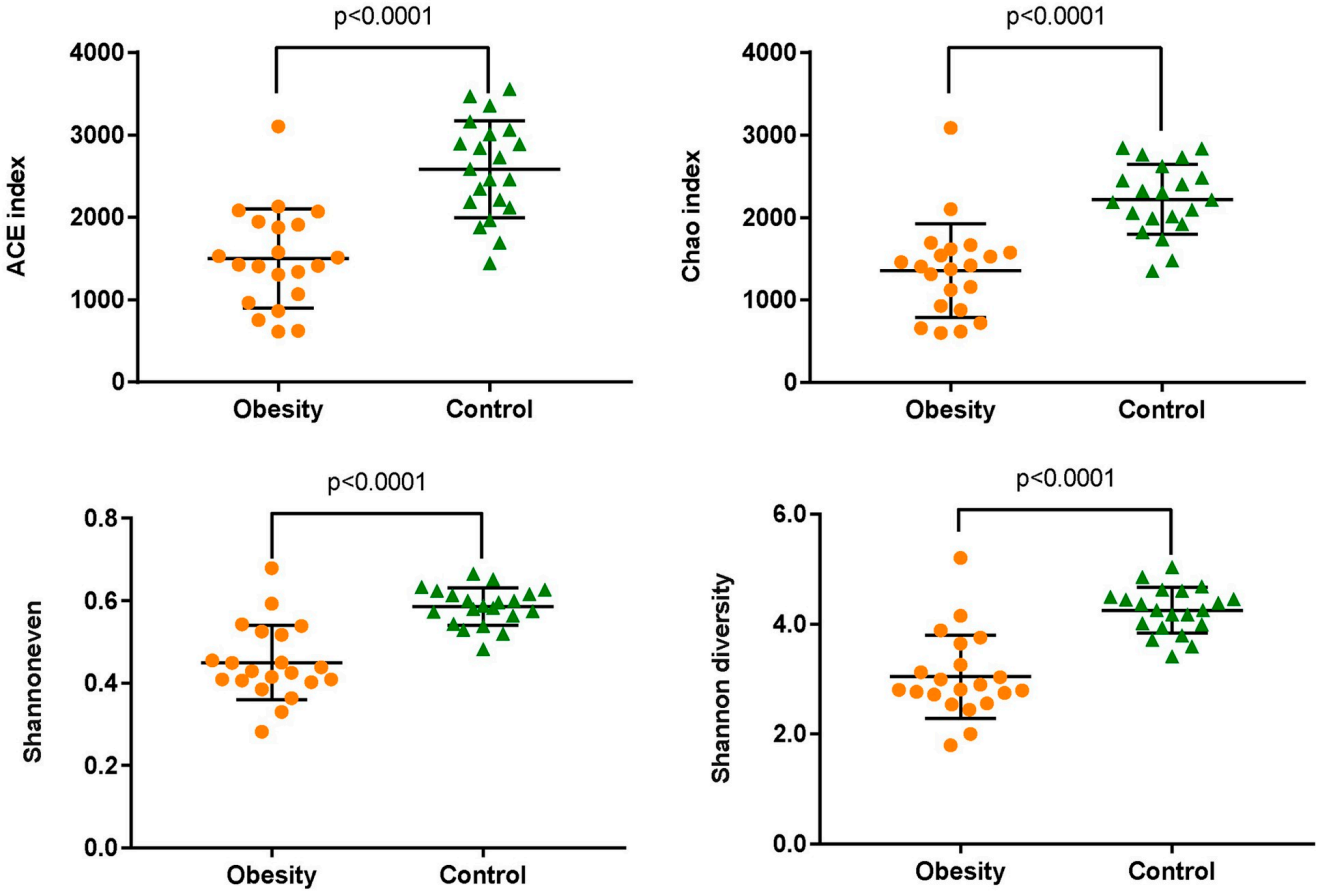
Comparison of bacterial richness, evenness and diversity between Obesity and Control groups.

According to the alpha diversity (Fig 2), obese adults showed lower richness (ACE index and Chao index), lower evenness (Shannon even index), and lower diversity (Shannon index), coinciding with the lower OTUs number observed in the Obesity group.

### Microbiota of Obesity and Control

**Fig 3:**
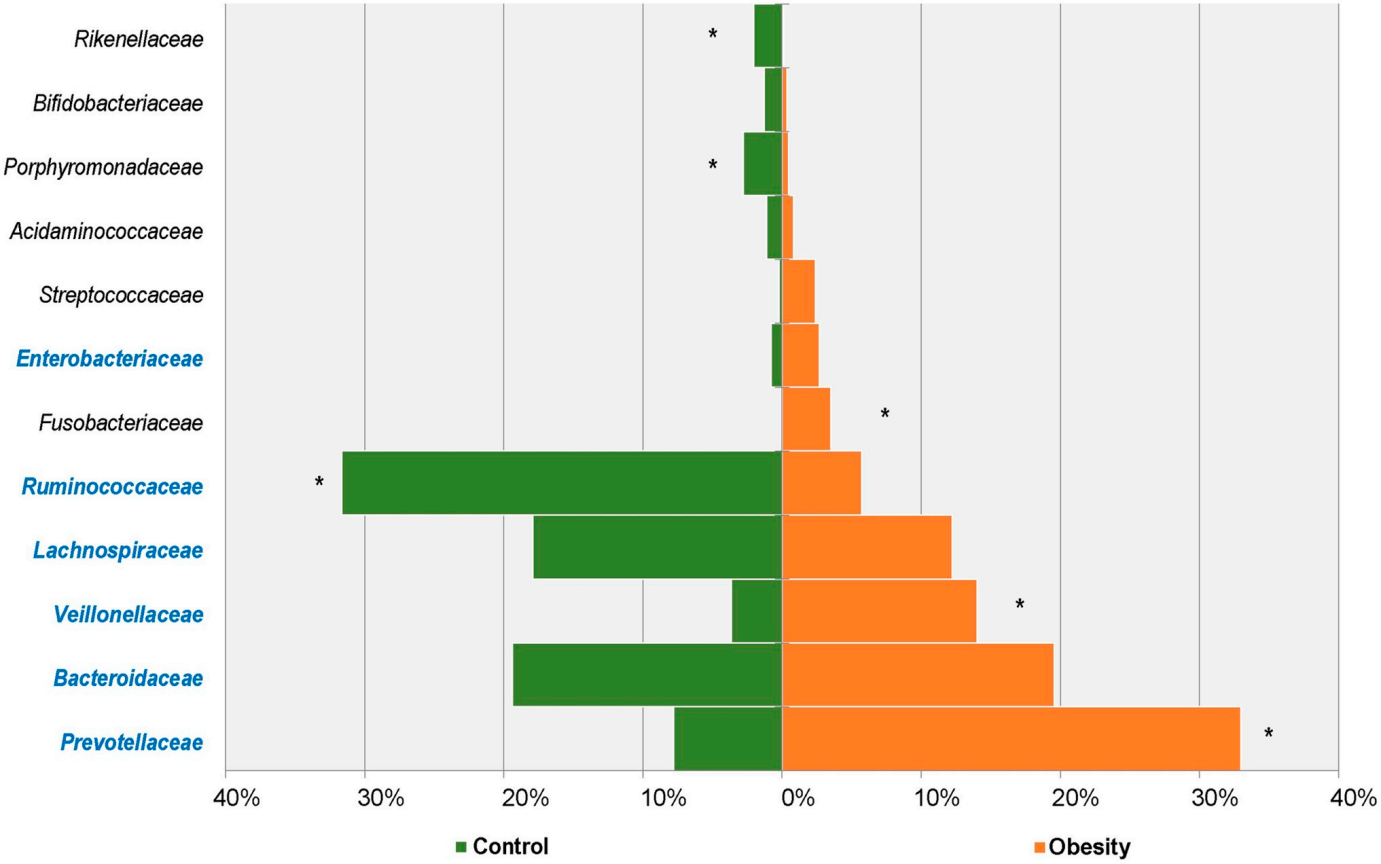
Major abundant families in Obesity and Control groups. The families with significant richness differences between the two groups were labeled with an asterisk.

**Fig 4:**
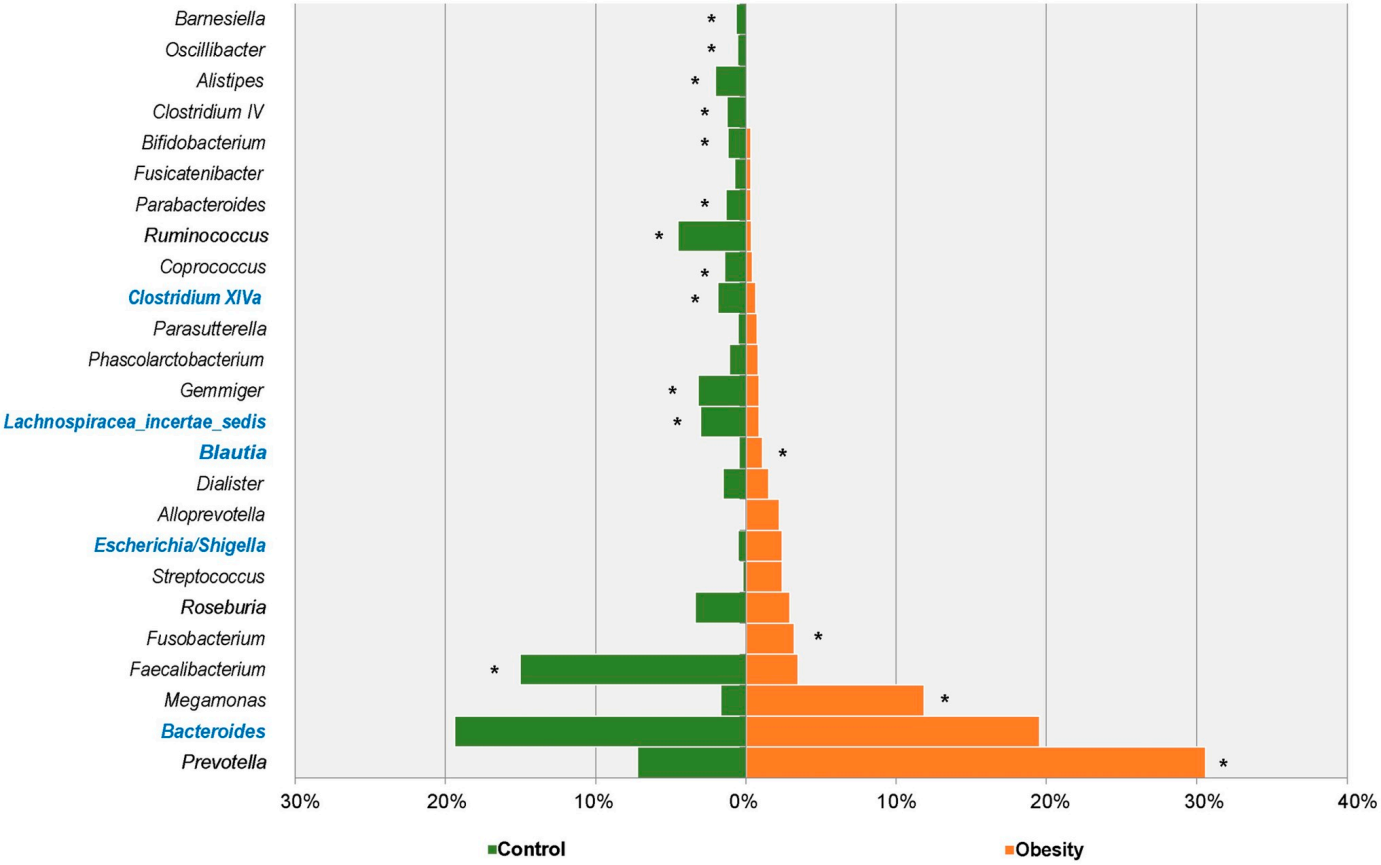
Major abundant genera in Obesity and Control groups. The genera with significant richness differences between the two groups were labeled with an asterisk.

The total gut microbiota was revealed through the phylogenetic and taxonomic assessments of the 16S rRNA V3-V4 regions. About 99.20% (±0.0014) of microbiota could be aligned to nine phyla, 94.92% (±0.0254) to 58 families and 84.04% (±0.0641) to 136 genera. At the phylum level, *Bacteroidetes* (average 43.12%, ±0.1069), *Firmicutes* (average 49.38%, ±0.1208), *Proteobacteria* (average 3.64%, ±0.0032) and *Fusobacteria* (average 1.78%, ±0.0176) were the four most abundant bacterial divisions in the gut, which were also the common phyla in all samples. At the family level, 12 families were most abundant in two groups (>1% in at least one group, accounting for over 87.55% in each group, Fig 3). Among them, *Lachnospiraceae*, *Ruminococcaceae*, *Veillonellaceae*, *Prevotellaceae* and *Bacteroidaceae* were dominant families (>80.38% of each group). In 136 identified genera, 25 genera were relatively abundant (>0.5% in at least one group, Fig 4), including *Bacteroides*, *Prevotella*, *Megamonas* and *Faecalibacterium*, etc. Among major genera, there were five ubiquitous (core) genera which were consistently found across all analyzed samples and comprised >24.63% of each group, that were genus *Bacteroides*, *Lachnospiracea_incertae_sedis*, *Clostridium XlVa*, *Escherichia/Shigella* and *Blautia*.

### Bacterial composition changes between Obesity and Control

**Fig 5:**
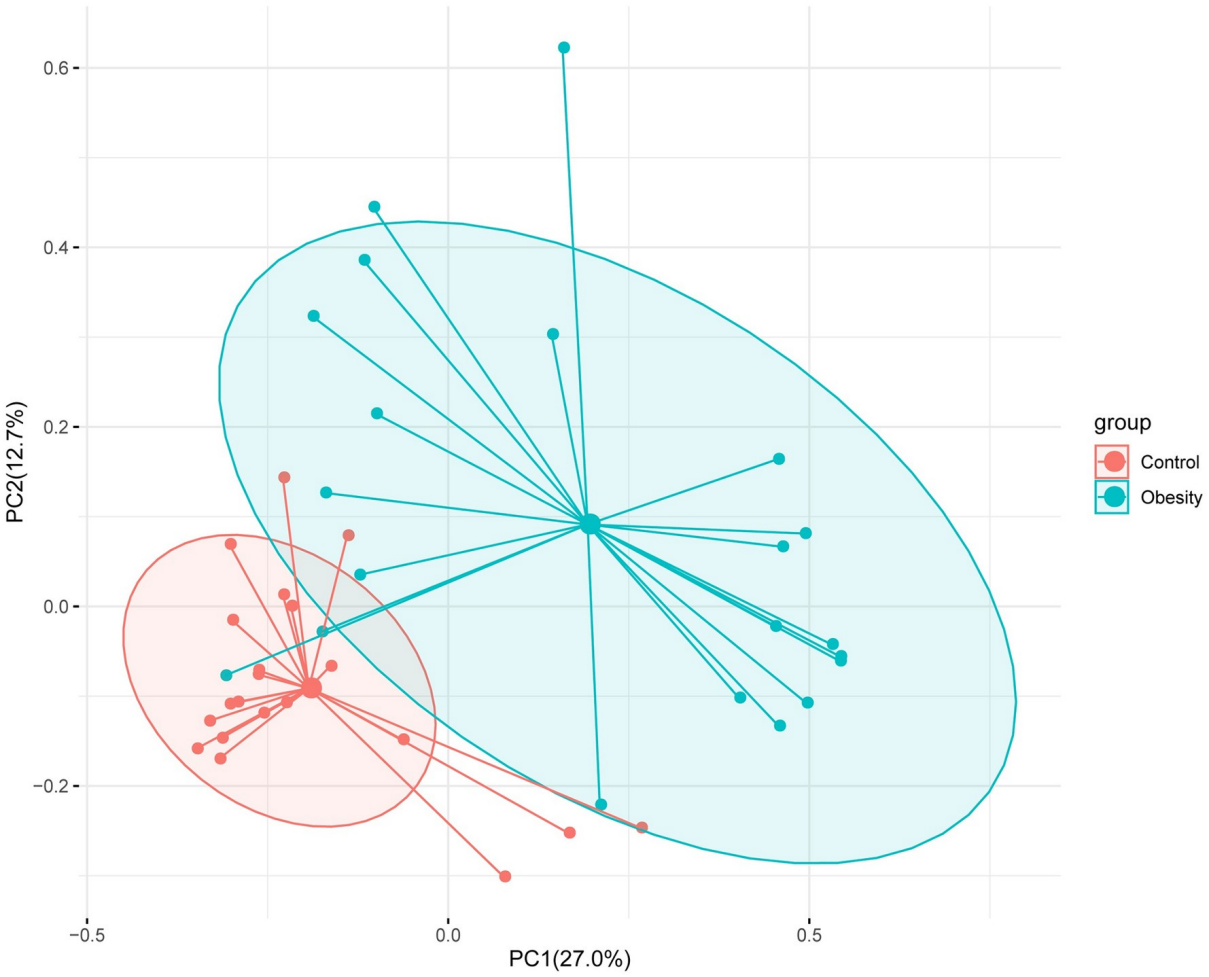
Principal co-ordinates analysis (PCoA) with Bray-Curtis dissimilarity based on genera.

AMOVA test showed a significant difference (*P*<0.05) between the Obesity and Control groups, and the PCoA analysis (Fig 5) also revealed that subjects of Obesity and Control were apart from each group based on the gut microbiota composition.

**Fig 6:**
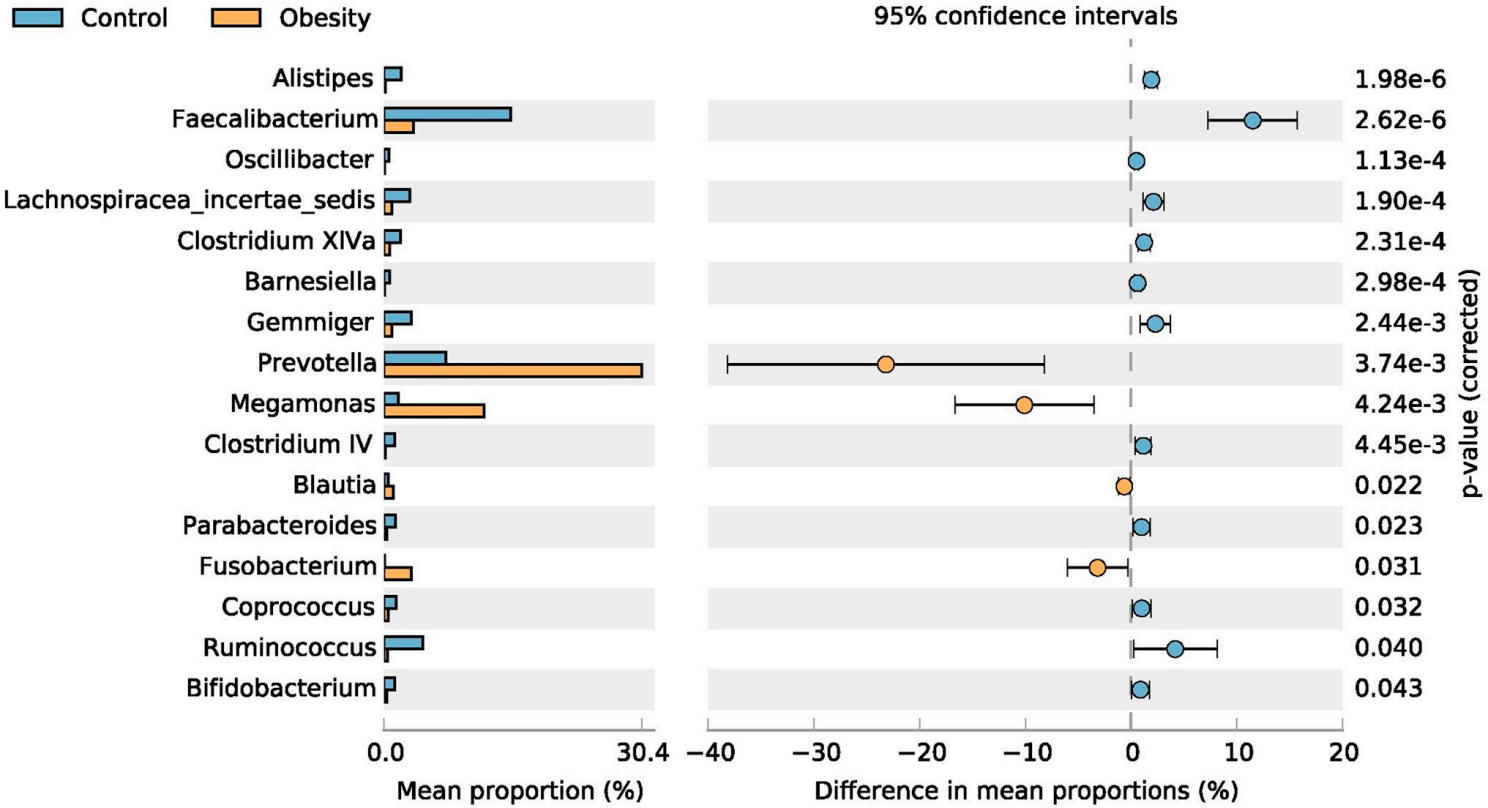
Gut microbiota comparison between obesity and control on the genus level. The genera with significant richness difference (*P* < 0.05, computed by STAMP) between the two groups were shown.

At the phylum level, four major abundant phyla showed significant variations between the Obesity (95.19%) and Control (95.70%) group, including *Bacteroidetes*, *Actinobacteria*, *Fusobacteria* and *Firmicutes* (Table 1). In our study, the abundance of *Firmicutes* decreased, while the abundance of *Bacteroidetes* increased in people with obesity. The ratio of Firmicutes/Bacteroidetes also decreased. At the family level (Fig 3), a total of six major families had significant difference between the Obesity (56.80%) and Control (47.92%) group. The six major families are *Prevotellaceae*, *Veillonellaceae*, *Ruminococcaceae*, *Fusobacteriaceae*, *Porphyromonadaceae* and *Rikenellaceae*, and three of them belong to *Bacteroidetes*. At the genus level (Fig 6), a total of 16 major genera had a significant difference between the Obesity (54.29%) and Control (45.52%) group, and 10 of them belong to *Firmicutes*. Of the 16 major genera, the abundance of four genera increased, while the remaining 12 decreased among the people with obesity. At the species level (OTUs from top 50, Table 2), we found nine abundant species had differences between the Obesity and Control group. Six of them were decreased in obesity gut microbiota, including *Faecalibacterium prausnitzii*, *Barnesiella intestinihominis*, *Alistipes putredinis*, *Bacteroides uniformis*, *Parabacteroides distasonis* and *Fusicatenibacter saccharivorans*. Three species were increased in obesity gut microbiota, including *Megamonas funiformis*, *Prevotella copri* and *Fusobacterium mortiferum*.

**Table 1:** Significantly different phyla of gut microbiota between the Obesity and Control groups.

phylum	Control: mean rel. freq. (%)	Control: std. dev. (%)	Obesity: mean rel. freq. (%)	Obesity: std. dev. (%)	p-values	Difference between means	95.0% lower CI	95.0% upper CI
Firmicutes	61.45	12.61	37.39	20.09	6.94E-05	24.06	13.28	34.85
Bacteroidetes	32.44	12.29	53.73	21.74	6.01E-04	-21.29	-32.67	-9.91
Actinobacteria	1.79	2.05	0.56	0.71	1.83E-02	1.22	0.23	2.22
Fusobacteria	0.02	0.08	3.51	6.57	2.75E-02	-3.49	-6.55	-0.43

**Table 2:** Significantly different species of gut microbiota between the Obesity and Control groups.

species	Control: mean rel. freq. (%)	Control: std. dev. (%)	Obesity: mean rel. freq. (%)	Obesity: std. dev. (%)	p-values	Difference between means	95.0% lower CI	95.0% upper CI
Faecalibacterium prausnitzii	13.34	6.65	2.96	5.05	2.40E-06	10.38	6.60	14.16
Barnesiella intestinihominis	0.53	0.51	0.03	0.09	2.90E-04	0.50	0.26	0.74
Alistipes putredinis	0.96	1.01	0.04	0.13	6.25E-04	0.92	0.44	1.39
Bacteroides uniformis	2.22	2.55	0.20	0.27	2.07E-03	2.02	0.83	3.21
Megamonas funiformis	0.79	1.83	8.45	10.31	3.59E-03	-7.66	-12.53	-2.80
Prevotella copri	5.74	11.97	24.86	25.18	4.71E-03	-19.12	-31.88	-6.36
Parabacteroides distasonis	0.62	1.03	0.09	0.10	3.22E-02	0.53	0.05	1.01
Fusobacterium mortiferum	0.02	0.08	2.84	5.81	4.22E-02	-2.82	-5.53	-0.11
Fusicatenibacter saccharivorans	0.64	0.62	0.27	0.49	4.57E-02	0.37	0.01	0.73

### Microbial co-abundance networks

**Fig 7:**
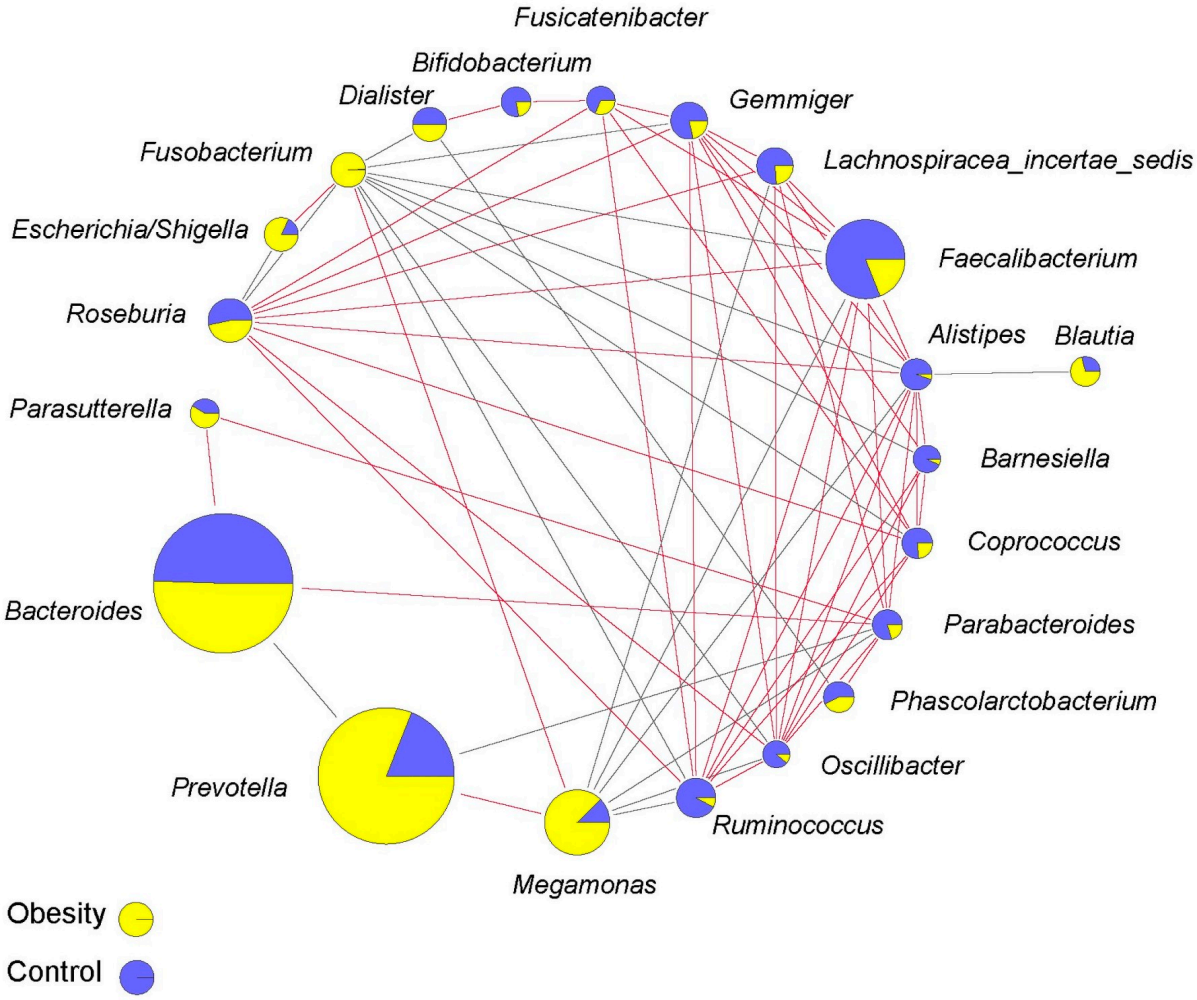
SparCC correlation networks observed between genera. The pie charts show relative genera proportions in the Obesity (yellow) and Control groups (blue), and the circle size represents the reads number. Line color: red (positive relationship) and grey (negative relationship).

To characterize the microbial interactions of gut microbiota, correlation patterns of the top 25 genera were calculated (Fig 7). In total, 48 positive and 20 negative significant correlations were found between 21 genera, of which *Blautia* showed only negative correlation while *Bifidobacterium*, *Fusicatenibacter* and *Parasutterella* showed only positive correlation with other genera.

### Predicted functional potential change between Obesity and Normal microbiome

**Fig 8:**
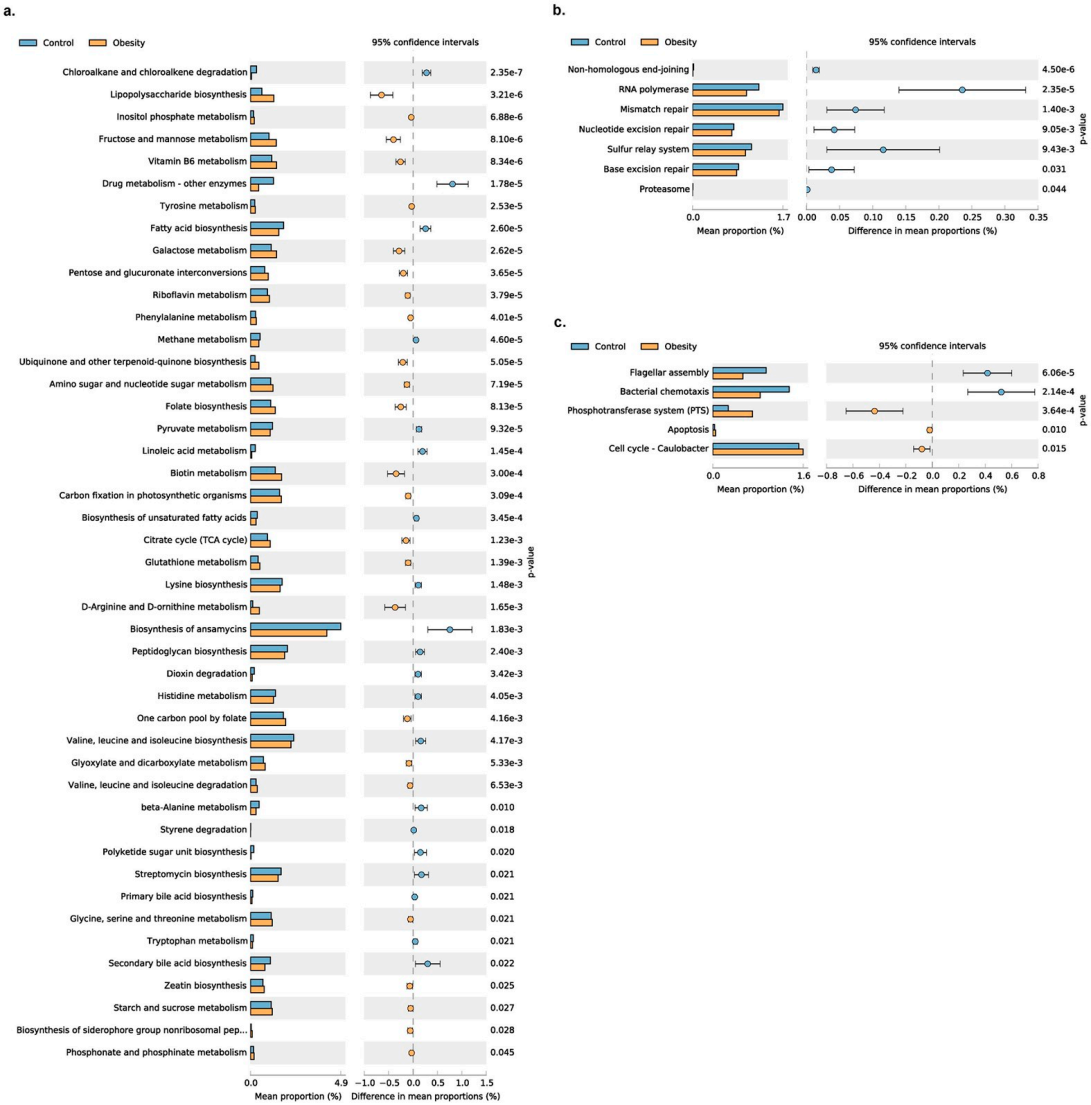
Predict the functional potential changes between obesity and control by using PICRUSt2. Panel a shows the changes of 45 pathways belonging to Metabolism between the Obesity and Control group. Panel b shows the changes of 7 pathways belonging to Genetic Information Processing between the Obesity and Control group. Panel c shows the changes of 5 pathways belonging to Environmental Information Processing and Cellular Processes between the Obesity and Control group.

We used PICRUSt2 to predict the functional potential changes for Obesity (Fig 8). A total of 57 pathways showed significant differences between the Obesity and Control group, with several pathways involved in Carbohydrate metabolism and sugar transport enriched in the Obesity group.

## Discussion

Gut microbes can influence obesity by regulating metabolism, homoeostasis, appetite and energy balance, which together play crucial roles in obesity [25]. The structure, function and diversity of the gut microbiota among people with obesity are different from those of normal people [26]. Individuals with obesity usually show lower biodiversity and richness [12]. Our results indicated that microbiota compositions were different between the Obesity and Control group, with the Obesity group showing lower diversity compared with the Control group (Fig 2). Many literatures have shown that obesity is related to the abundance of *Firmicutes* and *Bacteroidetes* and the ratio of *Firmicutes*/*Bacteroidetes*, but their changes are controversial in different studies [27–29]. The research of Ley RE et al. found that compared with normal people, *Bacteroidetes* are 50% lower in people with obesity, but their *Firmicutes* are higher, and losing weight by fat restricted or carbohydrate restricted dietary intervention can increase the abundance of *Bacteroidetes* [30]. In people with obesity induced by glucocorticoid (GC), the abundance of *Firmicutes* was increased and *Bacteroidetes* was decreased [31]. The research of Schwiertz A et al. showed that *Firmicutes* were significantly reduced in people with obesity, while *Bacteroidetes* were significantly increased, which led to a significant decrease in the ratio of *Firmicutes*/*Bacteroidetes* [27]. In our study, compared with normal subjects, *Firmicutes* of subjects with obesity were significantly decreased (control: 61.45%, obesity: 37.39%), while *Bacteroidetes* were significantly increased (control: 32.44%, obesity: 53.73%) (Table 1), and the ratio of *Firmicutes/Bacteroidetes* was decreased, which was consistent with the research results of Schwiertz A et al. [27]. The dispute about the changes of *Firmicutes* and *Bacteroidetes* may be related to the region, environment and diet, for example, people in the Shandong Province of China prefer food made of flour. At the family level, *Prevotellaceae*, *Porphyromonadaceae* and *Rikenellaceae* belong to *Bacteroidetes*. Among them, *Prevotellaceae* is increased in people with obesity, while the other two are decreased in people with obesity. *Veillonellaceae* and *Ruminococcaceae* belong to *Firmicutes*, and *Veillonellaceae* is increased in people with obesity, while *Ruminococcaceae* is decreased in people with obesity. Similarly, at the genus level, different genera belonging to the same phylum do not change in the same way. These may also be the reason why the changes of *Firmicutes* and *Bacteroidetes* are controversial in different studies.

The intestinal permeability and metabolites of people with obesity are different from normal people. High-fat diet reduces the expression of tight junction proteins including zonula occludens-1 (ZO-1) and occludin, thereby disrupting the integrity of the intestinal epithelium and increasing intestinal permeability [32,33].

SCFAs are metabolites of intestinal microbiota, which are generated through the fermentation of indigestible substances by gut microbiota [34]. SCFAs, primarily butyrate, propionate and acetate, can stimulate the release of the anorexigenic peptides including Peptide YY (PYY), amylin and Glucagon-like peptide 1 (GLP-1) and inhibit obesity caused by a high-fat diet. Among them, Butyrate significantly inhibits food intake [35]. Butyrate can also reduce intestinal permeability and improve intestinal barrier function by up-regulating tight junction protein expression [36].

The high concentration of SCFAs in feces is related to obesity, which may be caused by the low absorption efficiency of SCFAs [37]. The concentration of SCFAs in the feces of people with obesity is increased, while the higher concentration of SCFAs in feces is related to the lower gut microbiota diversity [37]. Therefore, the gut microbiota diversity of people with obesity is decreased, which is consistent with our conclusion. *Bacteroides uniformis* is negatively correlated with fecal butyrate, while *Blautia* and *Prevotella copri* are positively correlated with fecal butyrate and propionate [37]. In our study, the concentration of *Bacteroides uniformis* is significantly reduced while *Blautia* and *Prevotella copri* are significantly increased in individuals with obesity (Table 2), indicating that fecal SCFAs in the Obesity group is increased. The study of Lin H et al. had found that *Blautia* was positively correlated with deoxycholic acid (DCA), which was increased in rat with obesity on high-fat diet [38]. Increased DCA can promote obesity-associated diseases [39].

Our study showed that the abundance of *Bifidobacterium* is significantly reduced in the Obesity group (0.33%) compared to the Control group (1.21%) (Fig 6). *Bifidobacterium* can reduce obesity-related inflammation by restoring the balance of lymphocyte-macrophage, so it has an anti-obesity effect [40]. Other literature also reports that some species or strains of *Bifidobacterium* have anti-obesity effects [41], and the concentration of *Bifidobacterium* in the stool of individuals with obesity is significantly reduced [27]. Our results also showed that *Prevotellaceae* and *Veillonellaceae* were significantly increased in people with obesity. Serena C et al. reported that obesity is associated with increased levels of succinate produced by *Prevotellaceae* and *Veillonellaceae* which were increased in individuals with obesity [42]. In our study, a significant difference between the Obesity group (30.57%) and the Control group (7.22%) was found on *Prevotella*. Hu HJ et al. reported that *Prevotella* was positively correlated with triglycerides (TG) and high-sensitive C-reactive protein (hs-crp), and increased significantly in individuals with obesity [43]. Gao R et al. reported that among individuals with obesity, the beneficial bacteria such as *Bifidobacterium*, *Faecalibacterium* and butyrate-producing *Ruminococcaceae* are significantly reduced, while the potential opportunistic pathogens including *Fusobacterium* and *Escherichia/Shigella* are increased [44]. In our results, the changes of *Bifidobacterium*, *Faecalibacterium Ruminococcaceae Fusobacterium* and *Escherichia*/*Shigella* are consistent with the study of Gao R et al.

We predicted the functional potential changes between control and obesity using PICRUSt2. It can be seen that metabolic pathways have undergone significant changes in people with obesity, with pathways involved in carbohydrate metabolism and transport enriched in the the Obesity group, including Fructose and mannose metabolism, Galactose metabolism, Starch and sucrose metabolism, TCA cycle and PTS system, while pathways involved in Lipid metabolism reduced in the Obesity group (Fig 8).

The major limitation of this study is that the average age and geographical location are different between the two groups. The Obesity group was recruited from Jinan with an average age of 35 years, while the Control group was located in Beijing with an average age of 26 years. Since both Jinan and Beijing are located in the north of China, with a distance of about 410 km and sharing the same environment and eating habits, we ignored the impact of geographical differences on the gut microbiota. Meanwhile, the human gut microbiota is reported to be established in early life [45], and the composition is relatively stable throughout adulthood [46,47]. Based on this reason, we think the data from these two groups are comparable. Nevertheless, subsequent studies will consider the impact of these two factors and select samples with the same geographical location and ages.

## Conclusions

In our study, significant differences in microbiota between obesity and control subjects were revealed at different levels. The genus *Prevotella*, *Megamonas*, *Fusobacterium* and *Blautia* were significantly increased in subjects with obesity, while the genus *Faecalibacterium*, *Parabacteroides*, *Bifidobacterium* and *Alistipes* were significantly decreased. At the species level, we found that there were significant differences between the Control and the Obesity group in nine species, of which *Prevotella copri* was significantly increased in the obesity group. We also found that subjects with obesity have abnormalities in Carbohydrate metabolism. These findings provide support that gut microbiota can be used as target for treatment of obesity.
